# *Ginkgo biloba* extract (GbE) attenuates obesity and anxious/depressive-like behaviours induced by ovariectomy

**DOI:** 10.1038/s41598-020-78528-3

**Published:** 2021-01-08

**Authors:** Renata Mancini Banin, Meira Maria Forcelini Machado, Iracema Senna de Andrade, Lorenza Oliveira Testa Carvalho, Bruna Kelly Sousa Hirata, Heider Mendonça de Andrade, Viviane da Silva Júlio, Jéssica de Souza Figueiredo Borges Ribeiro, Suzete Maria Cerutti, Lila Missae Oyama, Eliane Beraldi Ribeiro, Mônica Marques Telles

**Affiliations:** 1 Disciplina de Fisiologia da Nutrição, Departamento de Fisiologia, Universidade Federal de São Paulo, Rua Botucatu, 862, Edifício de Ciências Biomédicas, 2º andar, Vila Clementino, São Paulo, SP CEP: 04023-062 Brasil; 2 Setor de Fisiologia e Farmacologia, Departamento de Ciências Biológicas, Universidade Federal de São Paulo, Diadema, SP Brasil

**Keywords:** Metabolism, Endocrine system and metabolic diseases, Anxiety, Depression

## Abstract

While several pieces of evidence link obesity and mood disorders in menopause, the mechanisms involved are not yet fully understood. We have previously demonstrated that *Ginkgo biloba* extract (GbE) both attenuated diet-induced obesity of male rats and restored serotonin-induced hypophagia in ovariectomized female rats. The present study aimed at exploring whether GbE treatment ameliorates ovariectomy-related obesity and anxious/depressive-like behaviours. Wistar female rats were either ovariectomized (OVX) or sham-operated (Sham). After 2 months, either 500 mg/kg of GbE or vehicle were administered daily by gavage for 14 days. Anxious/depressive-like behaviours were assessed by the Elevated Plus Maze and the Forced Swim Tests, respectively. Ovariectomy caused high visceral adiposity, hyperleptinemia, and hypercholesterolemia, and increased the anxiety index (*p* = 0.048 vs. Sham + GbE) while it decreased the latency to immobility (*p* = 0.004 vs. Sham). GbE treatment in OVX rats improved body composition, adiponectin levels and blood lipid profile. It also reduced the anxiety index (*p* = 0.004) and increased the latency to immobility (*p* = 0.003) of OVX rats. Linear regression analysis demonstrated that leptin (*p* = 0.047) and total cholesterol levels (*p* = 0.022) were associated with anxious-like behaviours while body adiposity (*p* = 0.00005) was strongly associated with depressive-like behaviours. The results showed that GbE therapy was effective in attenuating the deleterious effects of ovariectomy on body composition, lipid profile, and anxious/depressive-like behaviours. Further studies are warranted to better understand the therapeutic potential of GbE in menopause.

## Introduction

Although menopause is recognized as a natural and physiological state, its consequences deserve attention and an adequate care. Particularly, menopause-related obesity and depression are considered important health problems, which affect negatively women’s quality of life^1^.

Women have been shown to have a twofold higher risk of developing depressive or anxious disorders in comparison to men. It has been estimated that, mostly due to the hormonal fluctuations of menopause, at least 20% of women will present some depressive or anxious symptoms^2^. Depressive like-behaviours have been demonstrated in ovariectomized rodents^3,4^, which have also exhibited alterations involved in depression, such as decrement of serotonergic activity and elevation of hippocampal levels of inflammatory mediators^5^.

Obesity has been related to depression, physical inactivity and worsening of menopause troubles^6^. A link between obesity-associated inflammation and depressive symptoms has recently been proposed. Excessive body weight and increased adiposity result in hypertrophy, lysis and necrosis of adipose tissue leading to the recruitment of macrophages and the secretion of pro-inflammatory factors^7^. In a study with obese and non-obese individuals diagnosed with major depressive disorder (MDD), the boost of some cytokines such as TNF-alpha and C-reactive protein (CRP) seemed to be partially dependent on body mass index (BMI)^8^.

There is evidence that estrogen reduction might be involved in depression-related neuroinflammation. Ovariectomized rats have exhibited depressive-like behaviours associated with high hippocampal levels of interferon-γ, IL-6, Toll-like receptor-4, and pNF-κBp65, while serotonin levels were decreased^5^.

Moreover, important risk factors for obesity-associated cardiovascular diseases, including dyslipidaemia and leptin/adiponectin imbalance, have been reported in obese post-menopausal women and ovariectomized animals^9–11^. In overweight middle-aged women leptin resistance has been associated with impairment of mental health^12^. This agrees with the demonstrations of antidepressant and anxiolytic effects of the hormone administered to control rodents^13^ and to the behavioural impairment reported in animal models of leptin resistance^14,15^. It is thus likely that leptin resistance may be associated with depression and anxiety in obese people, although the participation of leptin in mental disorders is not fully established in humans^16^.

Adiponectin has also been associated with depression and anxiety disorders^17^. A meta-analysis has concluded that depressive patients have lower serum adiponectin levels in comparison to healthy subjects^18^. Obese people tend to present hypoadiponectinemia, which has been associated with an excessive production of proinflammatory cytokines, contributing to the neuroinflammation^19^.

Data from our laboratory have demonstrated that ovariectomized rats developed obesity, transient hyperphagia, and impairment of the anorexigenic response to serotonin, as well as reduction of serotonin extracellular levels in the medial hypothalamus. The treatment with *Ginkgo biloba* extract (GbE) for 14 days decreased food and energy intake and restored serotonin hypophagia. In addition, GbE enhanced serotonin levels in the medial hypothalamus and reduced hypothalamic serotonin transporter density. These results led us to suggest a potential therapeutic action of GbE on the regulation of food intake in post-menopausal women, preventing excessive weight gain and ameliorating serotonin hypophagia^20^.

Studying diet-induced obese rats, we have demonstrated both anti-oxidant and anti-inflammatory roles for GbE, along with improvement of insulin signaling in gastrocnemius muscle and retroperitoneal adipose tissue. GbE also had an anti-obesogenic effect and improved the lipid profile^21–24^.

Furthermore, GbE has been shown to modulate short and long-term memory and it has been indicated as an alternative treatment for psychiatric disorders such as anxiety, depression and schizophrenia^25,26^.

These data show that GbE represents a promising therapeutic agent for the treatment of the metabolic and psychological changes associated with menopause. Thus, the present study aimed at further investigating the effect of GbE therapy on both the obesity and the depressive/anxious-like behaviours induced by ovariectomy.

## Results

### GbE ameliorates anxious-like and depressive-like behaviours in OVX rats

**Figure 1:**
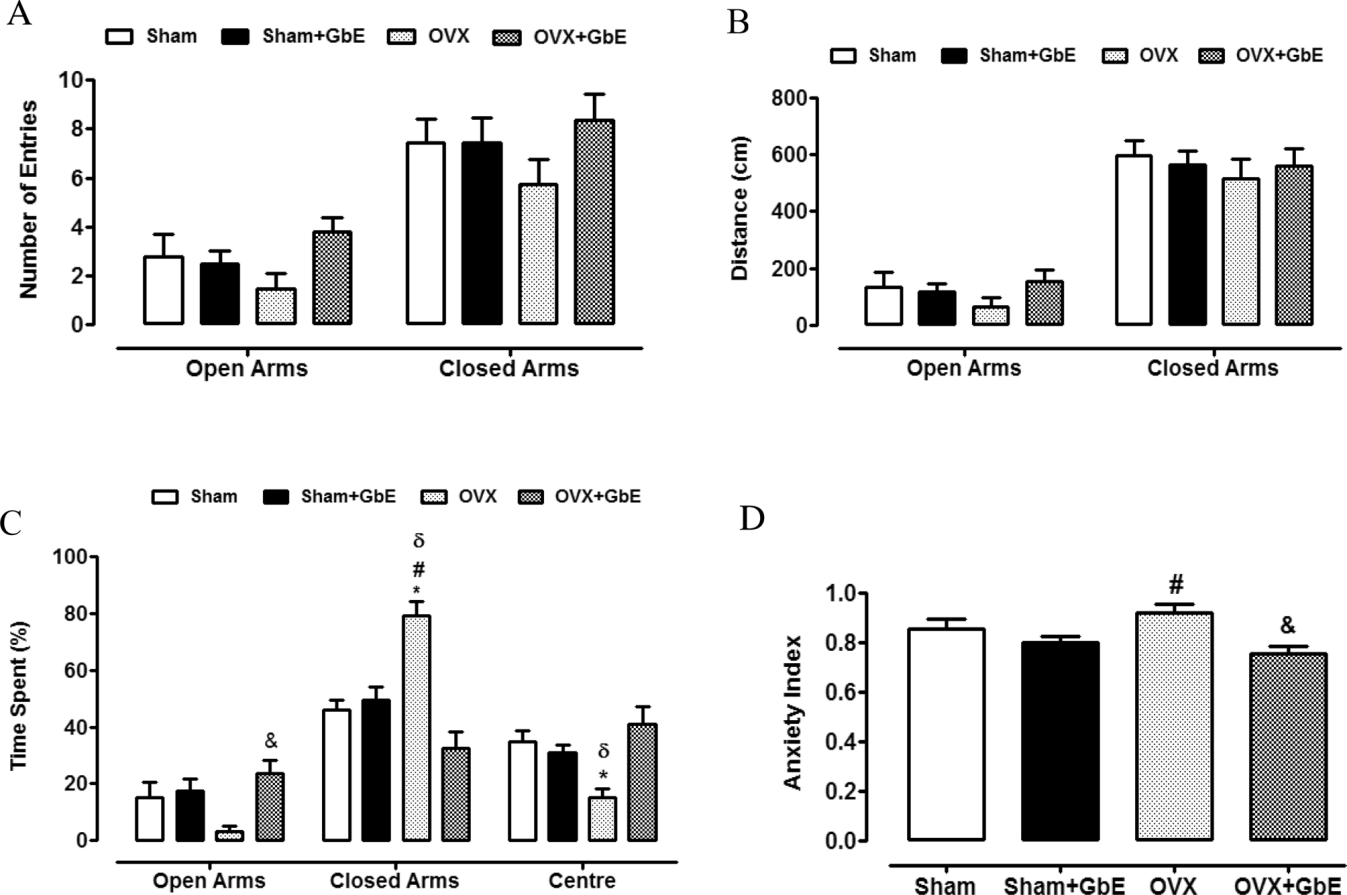
Anxious-like behaviours evaluated in the EPM test. (**A**) Number of entries, (**B**) travelled distance (cm), (**C**) time spent (%) in open and closed arms and (**D**) anxiety index of Sham (n = 8–9), Sham + GbE (n = 10–11), OVX (n = 10–11) and OVX + GbE rats (n = 9–11). *p ≤ 0.05 vs. Sham; ^#^p ≤ 0.05 vs. Sham + GbE; ^&^p ≤ 0.05 vs. OVX; ^δ^p ≤ 0.05 vs. OVX + GbE.

Figure 1 illustrates the number of entries (1A), distance travelled (1B), and percentage of time spent (1C) in open and closed arms and in the centre platform of the elevated maze, as measured in the EPM test, as well as the calculated anxiety index (1D). No differences among the groups were observed neither in the entries in the open [F_(3,38)_ = 2.060, *p* = 0.123] and in the closed arms [F_(3,41)_ = 1.200, *p* = 0.323], nor in the distance travelled in the open [F _(3,39)_ = 0.958, *p* = 0.423] and in the closed arms [F_(3,38)_ = 0.329, *p* = 0.805]. Concerning the percentage of time spent in the open arms [F_(3,40)_ = 4.212, *p* = 0.012], the OVX + GbE rats had a 333% increase in comparison to the OVX rats (*p* = 0.007). The OVX rats spent less time in the centre platform [F_(3,40)_ = 7.270, p = 0.001] in comparison to the Sham (− 56%, *p* = 0.012) and the OVX + GbE (− 63%, *p* < 0.0001) groups. In addition, OVX animals presented the highest percentage of time spent in the closed arms [F_(3,40)_ = 16.806, *p* < 0.0001] when compared to Sham (59%, p < 0.0001), Sham + GbE (60%, *p* < 0.0001) and OVX + GbE (143.5%, *p* < 0.0001) animals. Regarding the anxiety index [F_(3,39)_ = 5.087, *p* = 0.005], the OVX rats showed an index higher than that of the Sham + GbE rats (15%, *p* = 0.048) while the OVX + GbE group presented a lower index in comparison to the OVX group (− 17%, p = 0.004).

**Figure 2:**
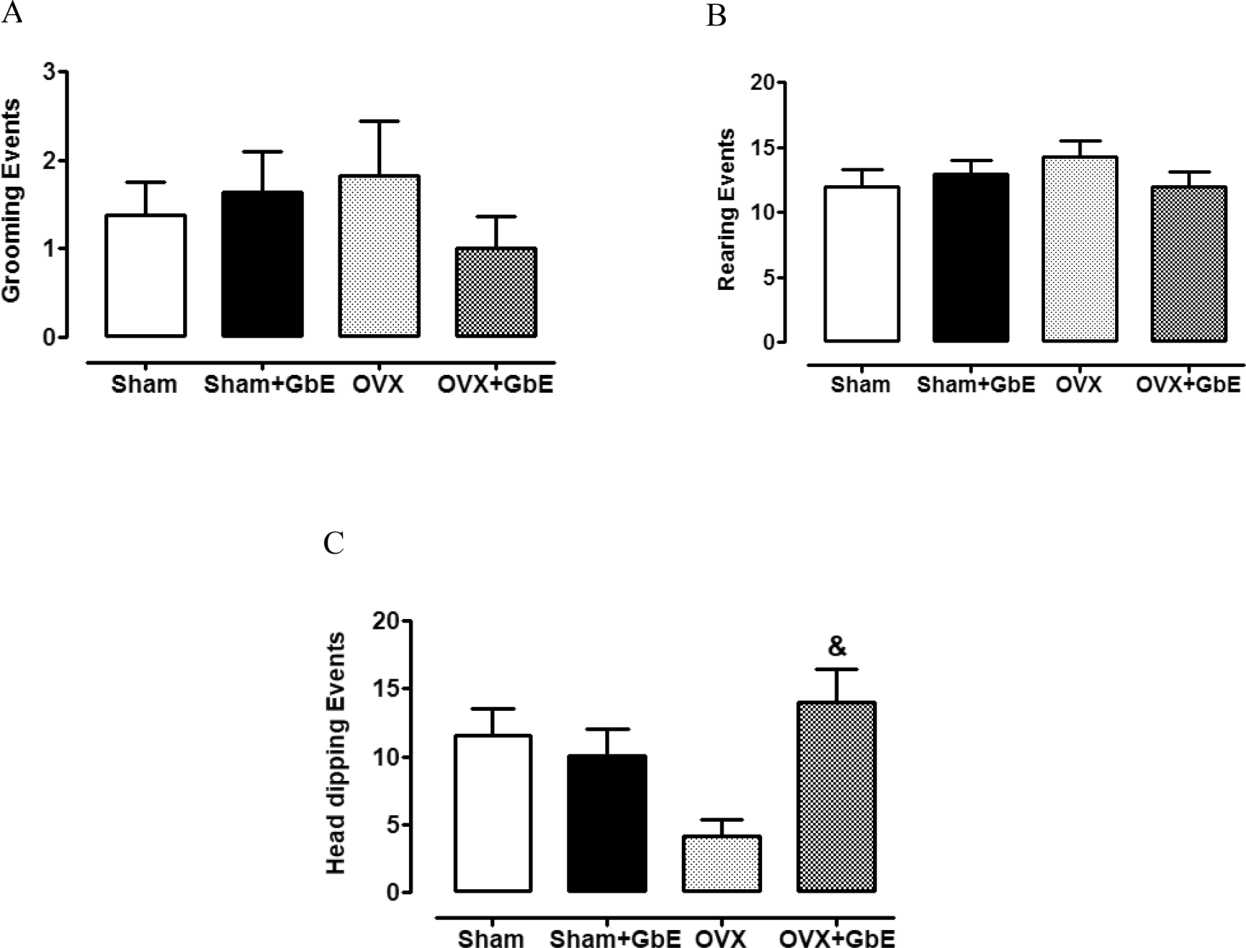
Anxious-like behaviours during the EPM test. Number of events of (**A**) grooming, (**B**) rearing and (**C**) head dipping during the EPM test of Sham (n = 8–9), Sham + GbE (n = 10–11), OVX (n = 10–11) and OVX + GbE rats (n = 9–11). ^&^p ≤ 0.05 vs. OVX.

The grooming (2A), rearing (2B), and head-dipping (2C) events are shown in Fig. 2. There was a significant elevation of the number of head-dipping occurrences [F_(3,41)_ = 4.814, *p* = 0.006] in the OVX + GbE group in comparison to the OVX group (235%, *p* = 0.004). No differences were observed in grooming [F_(3,40)_ = 0.580, *p* = 0.632] and rearing [F_(3,40)_ = 0.825, *p* = 0.489] events among the groups.

**Figure 3:**
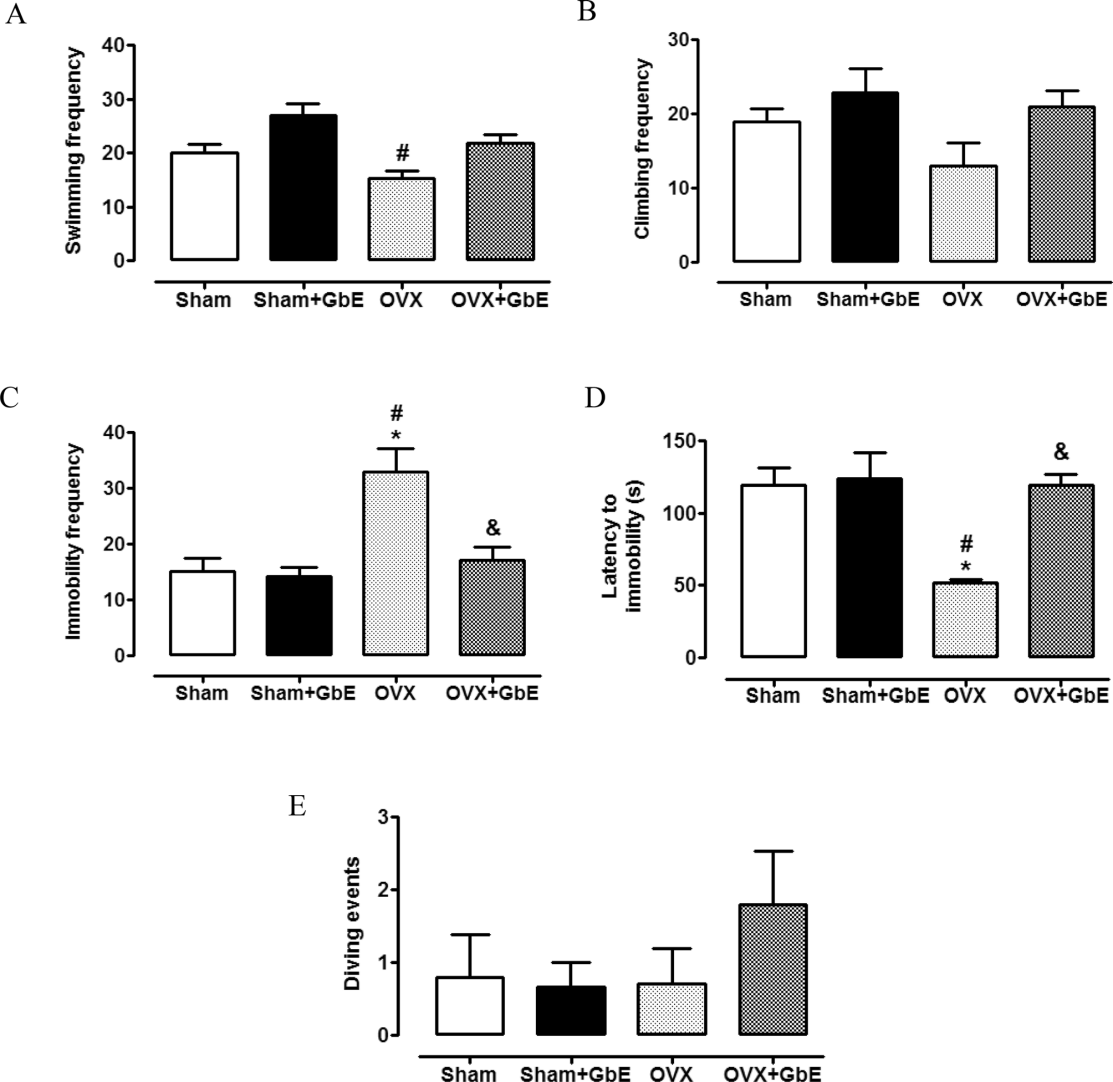
Depressive-like behaviours during the Modified Forced Swim Test. (**A**) Swimming, (**B**) climbing, (**C**) immobility frequencies, (**D**) latency to immobility and E) diving events of Sham (n = 8–9), Sham + GbE (n = 10–11), OVX (n = 9–10) and OVX + GbE rats (n = 9–10). *p ≤ 0.05 vs. Sham; ^#^p ≤ 0.05 vs. Sham + GbE; ^&^p ≤ 0.05 vs. OVX.

The results obtained in the Modified Forced Swim test are shown in Fig. 3. The OVX group demonstrated a lower swimming frequency [F_(3,37)_ = 7.777, *p* < 0.0001] than that of the Sham + GbE rats (43%, *p* < 0.0001). The OVX + GbE animals showed a non-significant increment of 42% (*p* = 0.075) in swimming frequency in relation to the OVX rats (Fig. 3A). Concerning the climbing frequency [F_(3,38)_ = 2.540, *p* = 0.072], represented in Fig. 3B, the OVX group presented a strong tendency of a 43% reduction in relation to the Sham + GbE group (*p* = 0.059). The OVX group presented a higher immobility frequency (Fig. 3C) [F_(3,37)_ = 10.125, *p* < 0.0001], in comparison to the Sham (117.5%, *p* = 0.001) and the Sham + GbE (132%, *p* < 0.0001) groups and a lower latency to immobility (Fig. 3D) [F_(3,38)_ = 7.423, *p* = 0.001] than those of the Sham (57%, *p* = 0.004) and the Sham + GbE (58%, *p* = 0.001) groups. The OVX + GbE rats showed a reduction of immobility frequency (− 48%, *p* = 0.002) as well as an increase in the latency to immobility (148%, *p* = 0.003), in comparison to the OVX group. The number of diving events was similar among the groups (Fig. 3E) [F_(3,37)_ = 0.958, *p* = 0.433].

### GbE improves body composition of OVX rats

**Figure 4:**
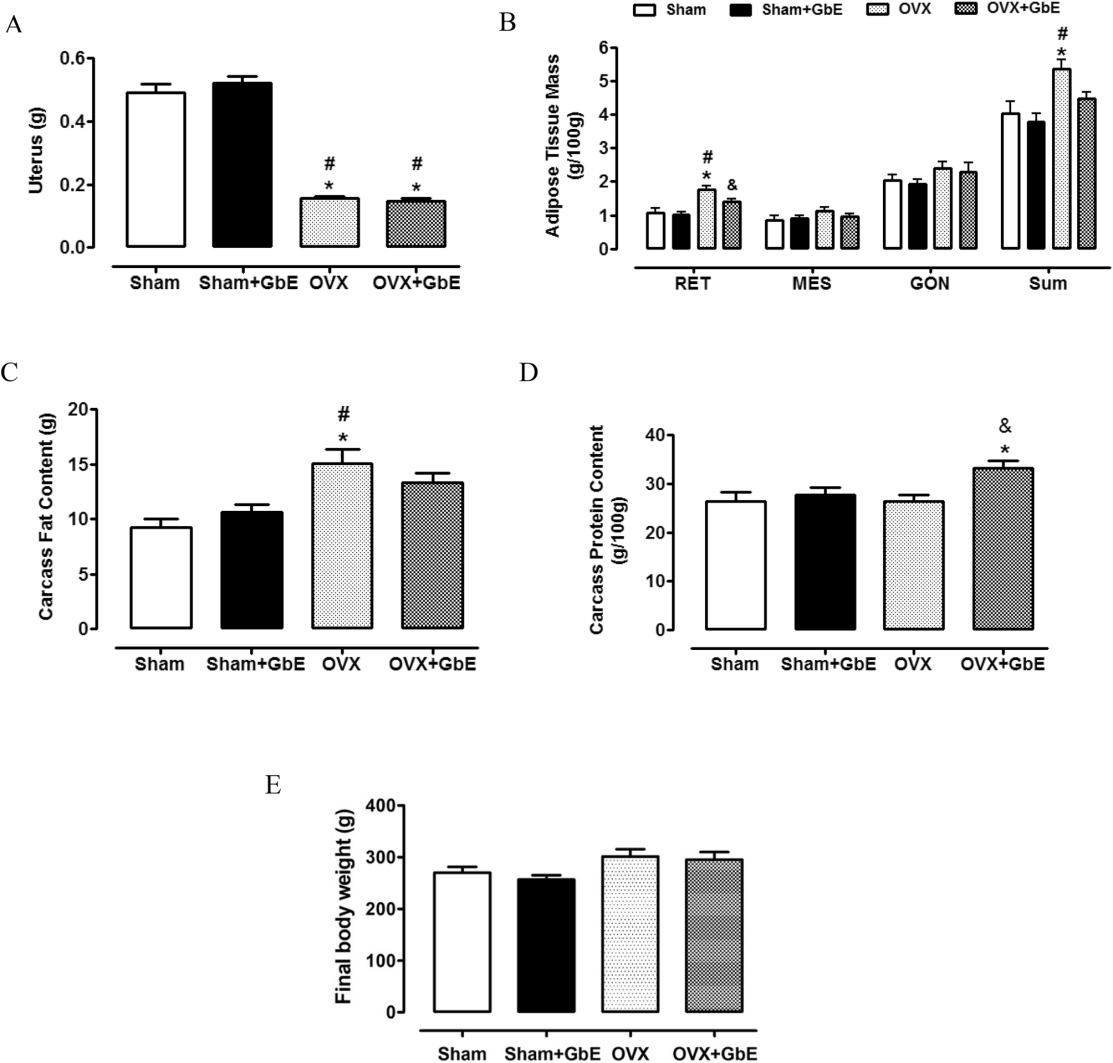
Body composition after GbE therapy. (**A**) Uterus (g) and (**B**) adipose tissue relative mass (g/100 g) of Sham (n = 10–11), Sham + GbE (n = 10–11), OVX (n = 10–11) and OVX + GbE rats (n = 10–13); (**C**) carcass fat (g) and (**D**) carcass protein content (g/100 g) of Sham (n = 5), Sham + GbE (n = 5–6), OVX (n = 7) and OVX + GbE rats (n = 5); (**E**) final body weight (g) of Sham (n = 8–9), Sham + GbE (n = 10–11), OVX (n = 9–10) and OVX + GbE rats (n = 9–10).*p ≤ 0.05 vs. Sham; ^#^p ≤ 0.05 vs. Sham + GbE; ^&^p ≤ 0.05 vs. OVX. *RET* retroperitoneal adipose tissue, *MES* mesenteric adipose tissue, *GON* gonadal adipose tissue.

The effectiveness of ovariectomy was confirmed by the significant uterus atrophy of OVX and OVX + GbE rats (F_(3,46)_ = 128.772, *p* < 0.0001) when compared to both Sham (*p* < 0.0001) and Sham + GbE (*p* < 0.0001) rats (Fig. 4A). Figure 4B depicts the elevated retroperitoneal fat pad [F_(3,45)_ = 13.395, *p* < 0.0001] presented by the OVX group, in relation to both the Sham (65%; *p* < 0.0001) and the Sham + GbE (72%; *p* < 0.0001) groups. GbE restored retroperitoneal adipose tissue mass of OVX + GbE, promoting a reduction of 21% (*p* = 0.033), in comparison to OVX rats. No differences were observed in relation to mesenteric [F_(3,48)_ = 1.674, *p* = 0.186] and gonadal [F_(3,46)_ = 1.194, *p* = 0.323] fat pads among the groups. A significant elevation of the sum of adipose tissues masses was observed in the OVX group [F_(3,45)_ = 6.459, *p* = 0.001] in relation to the Sham (33%, *p* = 0.006) and the Sham + GbE (41%, *p* = 0.001) groups. The OVX group exhibited 64% (*p* = 0.006) and 42% (*p* = 0.018) increases of carcass fat content [F _(3,21)_ = 6.552, *p* = 0.003], in comparison to the Sham and the Sham + GbE groups, respectively (Fig. 4C). Considering carcass protein content (Fig. 4D), the OVX + GbE group had an increment [F_(3,21)_ = 4.263, *p* = 0.019] of 26% (*p* = 0.034) and 25.5% (*p* = 0.022), in comparison to the Sham and the OVX groups, respectively. At the 14th day of the phytotherapy treatment, OVX rats tended to present a higher body weight [F_(3,21)_ = 3.046, *p* = 0.041] in relation to Sham + GbE group (*p* = 0.057), but GbE did not restore this parameter in the OVX + GbE group (Fig. 4E).

### GbE improves hormone levels and lipid profile of OVX rats

The measured serum parameters are illustrated in Table 1. No significant differences among the groups were observed in glycemia [F_(3,57)_ = 1.148, *p* = 0.338], fasting insulin levels [F_(3,43)_ = 0.678, *p* = 0.571], HOMA-IR [F_(3,44)_ = 0.417, *p* = 0.742], HOMA-β [F_(3,42)_ = 0.570, *p* = 0.638] and TNF-α levels [F_(3,44)_ = 0.076, *p* = 0.972]. However, leptin levels [F_(3,44)_ = 4.109, *p* = 0.012] were 139% higher in the OVX group in comparison to the Sham + GbE group (*p* = 0.006). Additionally, the OVX + GbE group presented an increment of 115% on adiponectin levels [F_(3,43)_ = 3.234, *p* = 0.032] in relation to the Sham group (*p* = 0.043).

**Table 1:** Serum parameters of Sham, Sham + GbE, OVX and OVX + GbE.

Serum Parameters	Sham	Sham + GbE	OVX	OVX + GbE
Glycemia (mg/dL)	130.49 ± 8.89	128.78 ± 7.71	137.94 ± 12.37	151.69 ± 10.24
Fasting insulinemia (ng/mL)	3.56 ± 0.69	3.86 ± 0.74	3.63 ± 0.64	2.66 ± 0.46
HOMA-IR	9.31 ± 1.73	11.45 ± 2.33	11.41 ± 2.75	8.56 ± 1.98
HOMA-β	58.92 ± 10.43	80.05 ± 18.92	68.30 ± 11.11	50.86 ± 8.01
Leptin (ng/mL)	15.55 ± 2.94	9.21 ± 1.09	**22.05 ± 3.68**^**#**^	15.88 ± 1.59
Adiponectin (ng/mL)	12.97 ± 1.75	16.30 ± 1.77	23.94 ± 4.45	27.95 ± 5.71*
TNF-α (pg/mL)	49.41 ± 2.18	46.64 ± 3.73	48.12 ± 2.86	40.94 ± 5.94

Serum parameters of Sham (n = 10–14), Sham + GbE (n = 11–16), OVX (n = 10–14) and OVX + GbE (n = 10–15) groups.

*p ≤ 0.05 vs. Sham; ^#^*p* ≤ 0.05 vs. Sham + GbE; ^&^p ≤ 0.05 vs. OVX.

**Figure 5:**
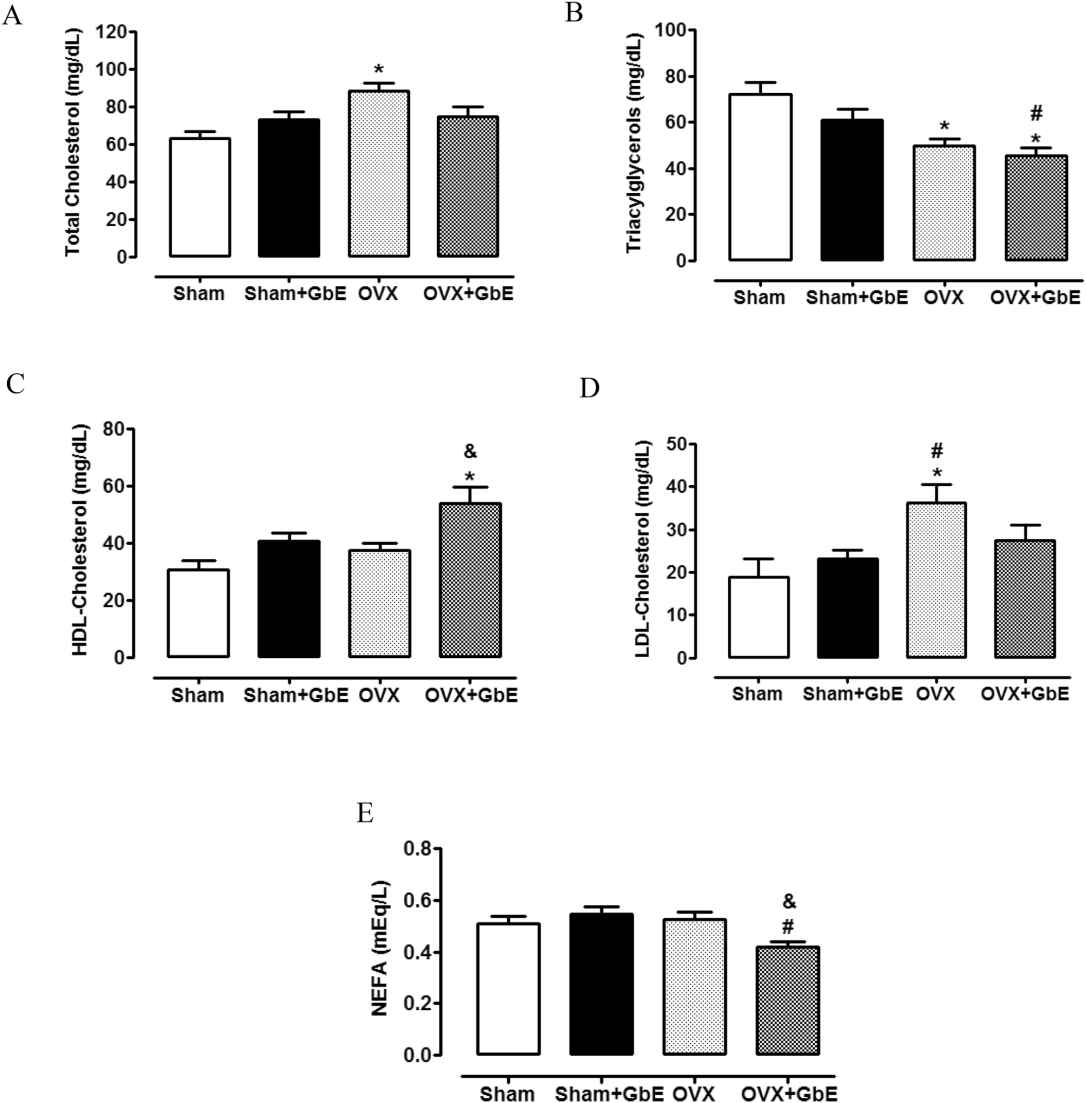
Lipid profile after GbE treatment. (**A**) Total cholesterol, (**B**) triacylglycerols, (**C**) HDL-Cholesterol, (**D**) LDL-cholesterol and (**E**) NEFA (mEq/L) of Sham (n = 10–11), Sham + GbE (n = 13–15), OVX (n = 11–13) and OVX + GbE (n = 12–15) groups. *p ≤ 0.05 vs. Sham; ^#^*p* ≤ 0.05 vs. Sham + GbE; ^&^*p* ≤ 0.05 vs. OVX.

In relation to the lipid profile (Fig. 5A–E), it can be noted that the OVX group presented hypercholesterolemia [F_(3,47)_ =  5.288, *p* = 0.003], as evidenced by an increase of 40.5% (*p* = 0.002) on total cholesterol levels when compared to the Sham group as well as an elevation of 92% (*p* = 0.015) and 57% (*p* = 0.044) on LDL-Cholesterol levels [F_(3,51)_ = 3.956, *p* = 0.013], in comparison to the Sham and the Sham + GbE groups, respectively. The treatment with GbE increased HDL-Cholesterol levels of the OVX + GbE group [F_(3,55)_ = 5.875, *p* = 0.002], by 75% (*p* = 0.001) in relation to the Sham group, and by 43% (*p* = 0.028) in relation to the OVX group. Ovariectomy caused a decrement [F_(3,54)_ = 7.944, *p* < 0.0001] of 31.3% of triacylglycerol levels, as compared to the Sham group (*p* = 0.004). The GbE therapy failed to modify this effect, leading to triacylglycerol levels 39% (*p* < 0.0001) and 26% (*p* = 0.046) lower than those of Sham and Sham + GbE animals, respectively. NEFA concentrations were 23.5% and 20.5% lower in the OVX + GbE [F_(3,55)_ = 4.439, *p* = 0.007] group in comparison to those in the Sham + GbE (*p* = 0.006) and the OVX (*p* = 0.045) groups, respectively.

### Body composition, hormonal, and metabolic parameters associate with anxious-like and depressive-like behaviours

The following parameters were included in the correlation analysis: body weight, retroperitoneal/mesenteric/gonadal adipose tissues masses, sum of fat depots masses, carcass fat and protein contents, all measured serum parameters and all measured parameters of the behavioural tests. Table 2 shows the variables with at least one significant correlation.

**Table 2:** Pearson’s correlations coefficients of the Sham, Sham + GbE, OVX and OVX + GbE groups.

	Climbing	Immobility frequency	Latency to immobility	Anxiety index	Open arms entrance	Open arms time spent	Open arms distance travelled	Closed arms time spent	Closed arms distance travelled	Head dipping
Body weight (g)	− 0.6638*	0.4753	− 0.6598*	0.1934	**− **0.3673	**− **0.1510	**− **0.3526	0.0179	0.6085*	**− **0.4055
Retroperitoneal Adipose Tissue (g/100 g)	− 0.6699*	0.7908*	**− **0.5715	0.4984	− 0.6106*	− 0.6035*	− 0.6040*	0.4212	**− **0.0112	− 0.6074*
Mesenteric Adipose Tissue (g/100 g)	**− **0.5878	0.2812	− 0.6490*	**− **0.1016	**− **0.0562	0.0437	**− **0.0641	**− **0.0697	0.4935	**− **0.2207
Sum of fat depots (g/100 g)	− 0.8324*	0.6142*	− 0.7128*	0.0680	**− **0.0728	**− **0.0560	**− **0.0130	0.0656	0.2474	**− **0.1818
Carcass fat (mg/100 g)	− 0.7130*	0.6751*	− 0.7332*	0.4365	**− **0.5064	**− **0.4200	**− **0.4541	0.3030	0.4237	**− **0.5607
Leptin (ng/mL)	**− **0.2367	0.2918	**− **0.2735	0.5873	**− **0.5255	**− **0.5021	**− **0.4519	0.6754*	**− **0.3293	**− **0.5150
Total cholesterol (mg/dL)	**− **0.0965	0.3199	**− **0.1092	0.5701	**− **0.5043	− 0.6694*	**− **0.4387	0.6012	**− **0.4193	**− **0.3471
HDL cholesterol (mg/dL)	0.2712	**− **0.5745	0.3579	− 0.6661*	0.4268	0.5198	0.2666	**− **0.4661	0.1053	0.4532
Uterus (g)	0.5939	**− **0.5938	0.6262*	**− **0.5886	0.6478*	0.4703	0.5822	**− **0.4185	**− **0.3096	0.5652

Pearson’s correlations between body/serum parameters and behavioural variables; n = 40; *p < 0.05 (two-tailed).

Considering depressive associated-behaviours evaluated by the forced swimming test, climbing events were negatively correlated with body weight (*p* = 0.026), retroperitoneal adipose tissue (*p* = 0.024), sum of fat depots (*p* = 0.001), and carcass fat (*p* = 0.014). The immobility frequency exhibited a positive correlation with retroperitoneal adipose tissue mass (*p* = 0.004), sum of fat depots masses (*p* = 0.044) and carcass fat (*p* = 0.023). The latency to immobility correlated negatively with body weight (*p* = 0.027), mesenteric adipose tissue (*p* = 0.031), sum of fat depots (*p* = 0.014), carcass fat (*p* = 0.010) and positively with uterus mass (*p* = 0.039).

In relation to anxiety related-behaviours evaluated in elevated plus maze, the anxiety index was negatively correlated with HDL-cholesterol levels (*p* = 0.025). The number of entries (*p* = 0.046), the distance travelled (*p* = 0.049) and the percentage of time spent in the open arms (*p* = 0.049) as well as the number of head dipping events (*p* = 0.047) were negatively correlated with retroperitoneal adipose tissue mass. In addition, the number of entries in the open arms was positively correlated with uterus mass (*p* = 0.031). The permanence in the open arms also showed a negative correlation with total cholesterol levels (*p* = 0.024). The percentage of time spent in the closed arms was positively correlated with leptin levels (*p* = 0.023) while the distance travelled in the closed arms demonstrated a positive correlation with body weight (*p* = 0.047).

Since the univariate analysis detected the existence of significant associations of body composition/metabolic/hormonal parameters and behavioural assessments denotative of anxious-like and depressive-like behaviours, multivariate linear regression models were constructed to identify the significant predictors for the depressive/anxious-like behaviours (Table 3).

**Table 3:** Linear regression models for anxious- and depressive-like behaviours as dependent variables.

Behaviour	Predictor	Beta coefficient	Standard error	*p* value	R^2^
Climbing	Body weight (g)	0.1011	0.0435	0.0258	0.1599
Immobility	Retroperitoneal adipose tissue (g/100 g)	19.2386	3.0986	< 0.0001	0.5171
Latency to immobility	Sum of fat depots (g/100 g)	− 23.8915	5.1630	0.00005	0.4824
Time spent in open arms	Total cholesterol (mg/dL)	− 0.28847	0.1211	0.0229	0.1431
Time spent in closed arms	Leptin (ng/mL)	0.81698	0.3974	0.0477	0.1135

For the climbing behaviour, the initial model tested body weight and sum of adipose tissues. The final model (*F*_(2,36)_ = 3.4284, *p* < 0.0433) indicated that climbing was positively associated with body weight while the sum of the fat depots showed no significant effect (p = 0.06520). Considering the immobility frequency, the model included only the retroperitoneal fat depot and it showed a significant positive association (*F*_(1,36)_ = 38.548, *p* < 0.00000). The latency to immobility was negatively associated with the sum of fat depots (*F*_(2,36)_ = 16.777, *p* < 0.00001). Body weight was also tested but it showed no significant association (*p* = 0.0860).

In relation to the percentage of time spent in the open arms, the final model (*F*_(2,34)_ = 2.8400, *p* < 0.07235) indicated a negative association with total cholesterol levels while the retroperitoneal fat pad mass had no significant association (*p* = 0.6226). The permanence in the closed arms (*F*_(1,33)_ = 4.2262, *p* < 0.04779) was positively associated with leptin levels.

## Discussion

The present findings indicated that ovariectomy caused anxious like-behaviours, as indicated by increased permanence in the closed arms and short permanence in the open arms, during the EPM test. The preference for the closed over the open arms has been attributed to spontaneous fear, which competes with the exploratory nature of rodents^27^.

The grooming behaviour, commonly observed when the animal is placed in an unfamiliar stressful situation, has been associated to obsessive–compulsive symptoms and considered as a measure of anxiety^28^. However, we failed to observe significant differences in this parameter among the groups.

In relation to depressive-like behaviours, the ovariectomized rats showed the highest frequency of immobility and the shortest latency to immobility, features highly indicative of a depressive-like state^29^. The present results agree with previous demonstrations of induction of anxious- and depressive-related behaviours by ovariectomy in rodents^4,30^ as well as of increased depression and anxiety rates in postmenopausal women^2,12^. Similarly, it has been reported, in both rats and mice, that estrogen replacement attenuated the increased immobility and decrease of active behaviours induced by ovariectomy^31^.

Anxiety and depression disorders are commonly treated with antidepressants that also have anxiolytic properties, such as sertraline, as well as with benzodiazepines. However, the long-term therapy with these drugs induce undesirable side effects, including sleep disturbances, body weight gain, sexual dysfunctions and dependence. Thus, the finding of more effective, safer and shorter-lasting therapies for the adequate control of these diseases is of great relevance^26^.

In the present study, GbE treatment was able to reduce the anxious-like behaviours of OVX rats, as indicated by a lower anxiety index and a higher number of non-protected head-dipping events. An anxiolytic role of GbE has been described in both animals and humans. In elderly patients diagnosed with mild cognitive impairment, daily treatment with 240 mg of EGb761 for 24 weeks reduced anxious symptoms in comparison to placebo^32^. In aged female rats, GbE (EGb761) oral supplementation (100 mg/kg, for 30 days) attenuated anxious-like behaviours while in young and middle-aged male mice, the same treatment caused a significant improvement of anxiety symptoms related to acute stress^33,34^.

The present results also indicated that the oral administration 500 mg/kg of GbE for 14 days promoted an antidepressant effect in ovariectomized rats. A similar effect has been observed in mice receiving an intraperitoneal dose of 10 mg/kg of EGb761 for 17 days. The authors associated the antidepressant action of EGb761 with an antioxidant effect in midbrain, prefrontal cortex and hippocampus^35^. The central mechanisms through which GbE affects mood and cognition have not been fully elucidated. Bilobalides present in GbE extracts have been shown to block long-term depression in rats, through increased synaptic plasticity in the medial perforant path-dentate gyrus (MPP-DG)^36^.

The present results data showed that ovariectomy induced increments of the retroperitoneal fat pad and carcass fat contents, in comparison to both the Sham and the Sham + GbE groups. In agreement, it has been reported that both OVX mice and rats developed overweight, with excess visceral and subcutaneous adipose tissues, which were related to increased adipocyte size and pre-adipocyte differentiation, as result of hypoestrogenism^37^. Similarly, postmenopausal women have been shown to present increased total and abdominal fat masses and reduction of lean body mass, resulting from the estrogen fall allied to a raise of testosterone levels^9^.

In the present study, ovariectomy induced hyperleptinemia, probably as a consequence of increased adiposity. It is well established that obesity-related hyperleptinemia is associated to leptin resistance, which leads to hyperphagia and decreased energy expenditure^38^. In premenopausal women, estrogens levels have been closely related to leptin levels and regulation of leptin receptors expression and sensitivity. Additionally, both post-menopausal women and ovariectomized animals presented an elevation of serum leptin and a reduction of adiponectin levels, what may promote insulin resistance^38,39^.

Although the ovariectomized rats of the present study exhibited higher blood levels of leptin, no signs of insulin resistance were observed. In ovariectomized mice, glucose intolerance developed only when the animals were exposed to a high-fat diet^40^. In another study, the authors noted that both diet-induced obesity and ovariectomy were able to lead to a state of insulin resistance in rats. However, those rats were submitted to ovariectomy at the age of 13 weeks, i.e., 5 weeks later than in our study^41^.

The present data also showed that GbE improved body composition, as it decreased retroperitoneal fat pad mass and protected against adipose tissue accumulation in ovariectomized rats. In addition, GbE raised adiponectin levels of OVX animals, what may have been a consequence of the lower adiposity, since this adipokine secretion is inversely proportional to body fat^11^. Corroborating the present findings, it has been reported that supplementation with *Morus alba* extract, an anti-inflammatory and anti-oxidant herbal medicine, to female rats fed a high-cholesterol diet, reduced body adiposity, up-regulated adiponectin gene expression, down-regulated leptin and resistin gene expressions, and improved insulin sensitivity in the visceral adipose tissue^42^.

In agreement with the present data, we have previously demonstrated, in obese rats, that GbE reduced both body weight and adiposity, epididymal adipocytes volume and incorporation of fatty acids into triglycerides, what allowed us to suggest a potential use of GbE in the control of obesity^22–24^. *Ginkgo biloba* biflavones have been found to exert a lipolytic action in the epididymal adipose tissue of rats, through inhibition of cAMP-phosphodiesterase^43^.

Decline in estrogens production has been associated with changes in lipid profile. The dyslipidaemia after menopause is characterized by high levels of TC and LDL-cholesterol, followed by decreased HDL-cholesterol levels, what may accelerate atherosclerotic processes^39^. In the present study, high levels of TC and LDL-cholesterol were observed in the ovariectomized rats, in agreement with a previous report^44^. However, TAG levels were decreased in both OVX and OVX + GbE rats, probably due to estrogen deficiency. In fact, ovariectomy has been demonstrated to impair the lipolytic response to norepinephrine in fat cells, as well as to increase the activity of adipose tissue lipoprotein lipase (LPL), mechanisms potentially linked to the increased fat deposition associated with ovariectomy^45^.

Importantly, GbE promoted a healthier lipid profile in ovariectomized rats, increasing HDL-cholesterol levels while protecting against the elevation of TC and LDL-cholesterol levels. These findings agree with our previous study showing GbE to decrease serum TAG and tending to decrease TC of obese male rats^22^. In addition, we observed a decrement of NEFA serum levels in OVX + GbE rats. It is well established that the elevation of blood NEFA may stimulate inflammatory pathways by activation of toll-like receptor 4, leading to an impairment of insulin sensitivity^46^. A protective role of GbE has also been demonstrated on the lipid profile alterations induced by ethanol intake and diabetes in rats^47,48^.

Obesity, increased visceral fat, insulin resistance and dyslipidaemias are components of the metabolic syndrome, whose prevalence becomes potentially high in post-menopausal women^9^. In addition, due to the gonadal hormones fluctuations from menarche until menopause establishment, women exhibit a higher vulnerability to develop anxiety and depression than men^1,2^. Although hormone replacement therapy (HRT) shows good results for the treatment of these menopause-associated problems, HRT is known to increase the risk of developing cardiovascular diseases, breast cancer and other tumors^9^.

In human breast cells in culture, GbE blocked the cell proliferation induced by high estrogen levels, presenting a chemoprotective role^49^. Considering the beneficial actions of GbE, it might be suitable to prevent menopause-related disturbances, without inducing breast cancer, the main unwanted effect of HRT.

A link between obesity and psychological diseases has been described. Excess visceral adiposity and unbalanced feeding habits have been implicated in the development of depressed mood during obesity establishment. In addition, depressive individuals are more prone to weight gain due to poor food choices and physical inactivity^50^. In American men and women, obesity has been highly associated with depression and anxiety^51^.

Here, we performed linear regression analyses to determine whether body composition and serum parameters might be predictors for the anxious/depressive-like behaviours associated with menopause. The data showed that leptin and total cholesterol were positive predictors for anxious-like behaviours. In agreement with the present findings, the hypercholesterolemia and hyperleptinemia of obese female rats have been shown to be positively associated with mood and anxiety symptoms. Interestingly, this study also reported a reduction of depressive- and anxious-like behaviours by the oral supplementation with *Morus alba* extract^42^.

The sum of fat depots was strongly associated with depressive-like behaviours, in agreement with the proposition of an influence of visceral adiposity on the development of depression^52^. In fact, even in healthy individuals, inadequate eating patterns have been linked to psychological disturbances^53^. Considering these observations, it seems reasonable that a relation of body adiposity gain and depression exists in both eutrophic and obese animals and humans.

It has been reported that obese individuals had a 55% higher risk of developing depression while depressed individuals presented a 58% higher chance of becoming obese. Excessive release of pro-inflammatory cytokines from increased visceral fat may induce depressive symptoms through the activation of hypothalamus–pituitary–adrenal axis, generating hypercortisolaemia and more fat deposition^54^. Maintenance of healthy body weight reduced depressive disorders in post-menopausal women^55^.

Finally, it has been reported that central inflammation may trigger behavioural disturbances, such as anxiety and depression, in menopausal women^56–59^. Despite the fact that TNF-alpha was similar among the groups, we cannot rule out the possibility of ovariectomy-induced inflammation, as observed by other authors^5^. The present lack of a statistically significant difference in TNF-alpha levels may have been a consequence of the short period after ovariectomy or of the age of the rats at the moment of euthanasia. In addition, other pro-inflammatory cytokines may be altered, as increased levels of IL-6 and of its soluble receptor have been reported in menopausal women with depressive mood^56^.

The present data showed that GbE reduced body adiposity and increased carcass protein and serum adiponectin levels in the ovariectomized rats, while it ameliorated anxious- and depressive-like behaviours. Thus, the present data suggest a potential for GbE to ameliorate menopause-related obesity and mood disorders. However, clinical studies are necessary to evaluate if the effects observed in ovariectomized rats are reproducible in menopausal women.

## Methods

### Animals

**Figure 6:**
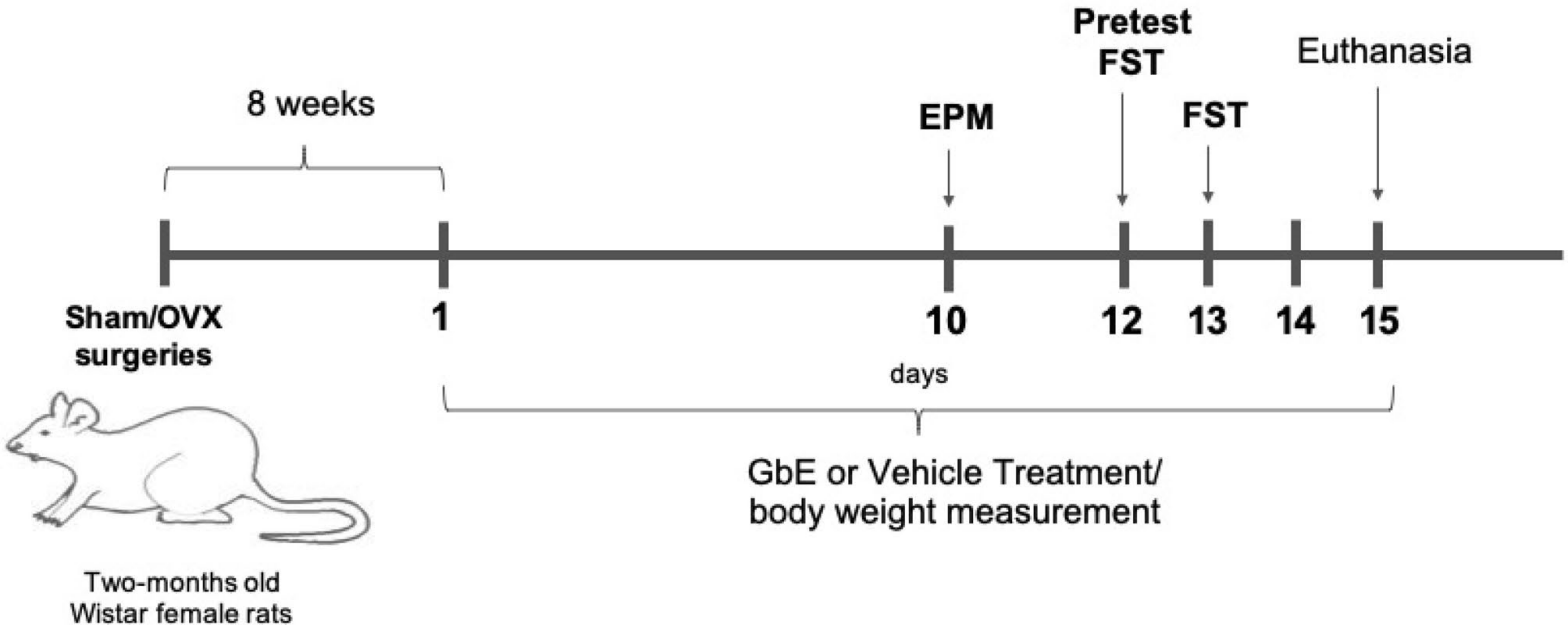
Experimental timeline.

All the procedures were approved by the Ethics Committee on Animal Research of the Universidade Federal de São Paulo and were performed in accordance with the principles of the Brazilian guideline for the use of animal models in research (Arouca Law—number 11794/08). Fifty nine 8-week-old female Wistar rats undergone bilateral ovariectomy (OVX) or false-ovariectomy (Sham) as previously described^20^. The survivor rate after surgical procedure was 100%. Eight weeks after surgery, the Sham and OVX rats were sub-divided according to the phytotherapy treatment described below. Figure 6 exhibits the experimental timeline.

### Phytotherapy treatment

Eight weeks after OVX or Sham operation, the animals were subdivided in two groups which received, for 14 days (once daily, by gavage), either 1.5 mL of saline (Sham and OVX groups) or 500 mg/kg of standardized extract of *Ginkgo biloba* (GbE) diluted in 1.5 ml of saline (Sham + GbE and OVX + GbE groups). During the phytotherapy treatment, body weight was measured daily.

The GbE extract (Huacheng Biotech Inc., China) contained flavone glycosides (25.21%), terpenoids (6.62%), ginkgolides A, B, C (3.09%) and bilobalides (2.73%).

### Behavioural evaluation

During the phytotherapy treatment, the animals were submitted to the Elevated Plus Maze test (EPM) to evaluate anxious-like behaviours and to the Modified Forced Swim test (FST) in order to identify depressive-like behaviours. A two-day interval was allowed between the tests.

#### Analysis of anxious-like behaviour: EPM test

The plus-maze apparatus was made of wood and positioned 50 cm above the floor. It contained two closed arms (CA, 10-cm wide and 40 cm high walls) and two open arms (OA, 10-cm wide, without walls) connected by a central platform, as previously described^27,60^. All tests were conducted during the light phase of the light/dark cycle, between 9 am and 2 pm. All sessions were recorded for subsequent analysis.

At the 10th day of the gavage treatment, the animals were individually placed in the central platform, facing an open arm, and allowed 5 min to explore the apparatus.

The percentage of time spent in the OA [% time in OA = (time spent in the OA /time spent in all arms) × 100)] and the percentage of entries into the OA [% entries in OA = (OA entries/total entries in all arms) × 100] were measured to evaluate anxious-like behaviour. In addition, the total number of entries (into both enclosed and open arms) was used to evaluate spontaneous locomotor activity^61^.

The number of risk assessments, which consists of stretch-attend postures, head-dipping, grooming and rearing were used as complementary measures, since they have been used to determine when rodents evaluate and/or avoid the OA^60,62^. The anxiety index was calculated as: 1 − [(OA time (min)/5 min) + (number of OA entries/total entries)]/2. The results range from 0 (less anxious) to 1 (more anxious)^63^. The maze was thoroughly cleaned between subjects.

#### Analysis of depressive-like behaviour: FST

At the 12th and 13th days, each animal was placed in a transparent cylinder, measuring 30 cm in diameter and 50 cm in height, filed with water (24 ± 1 °C) up to 30 cm, to avoid both the touching of the bottom and the escaping from the tank^31^. All animals were subjected to two sessions. At the 12th day, a 15-min. training session was carried out, for acclimation to the testing environment. After 24 h, the 5-min test was performed. The latency to immobility was used as an indication of behavioural despair^29^. The animal was considered immobile when making only necessary movements to keep its head above the water or floating^3,29^. The frequency of immobility, and the number of swimming, climbing, and dip events were counted every five seconds^31,63^.

### Serum parameters and mass of peripheral tissues

The overnight-fasted rats were anesthetized with thiopental (80 mg/kg, i.p.) before euthanasia. Trunk blood was collected, centrifuged (1258 g, 15 min., 4 °C), and the serum was stored at – 80 °C until analyses. Uterus and retroperitoneal, gonadal and mesenteric fat pads were dissected and weighed. Ovariectomy was confirmed by total uterus atrophy, contrasting with Sham rats. Glucose, TAG, TC, and HDL-cholesterol serum levels were determined by enzymatic colorimetric methods using commercial kits (Labtest Diagnóstica, Brazil) and NEFAs serum levels were determined by a commercial kit (Wako Pure Chemical Industries, Japan). Serum insulin, leptin, adiponectin and TNF-α levels were measured using a Milliplex kit (Millipore, USA). LDL-cholesterol concentration was determined by the F. Friedewald calculus: LDL-Cholesterol (mg/dL) = (TC (mg/dL) − HDL-Cholesterol (mg/dL)) − (TAG (mg/dL)/5). The homeostasis model assessments for insulin resistance (HOMA-IR) and beta-cell function (HOMA-β) were calculated from fasting insulin (mU/mL) and fasting glucose (mmol/L), as follows: HOMA-IR = (insulin × glucose)/22.5; HOMA-β = (insulin × 20)/(glucose × 0.0555) – 3.5.

### Carcass fat and protein contents

After the removal of the tissues (gastrointestinal tract, liver, uterus, and retroperitoneal, gonadal and mesenteric fat pads), the carcasses were kept in freezer – 20 °C until analyses. For the determination of carcass lipid and protein contents, carcasses were shaved, softened, homogenized and digested in 30% potassium hydroxide and 6 N sulfuric acid. Lipid was extracted from 5 g aliquots with petroleum ether and quantitated gravimetrically^64,65^. Protein from 2 g aliquots was dissolved in KOH 0.6 N and quantified according to Bradford method (Bio-Rad, Hercules, CA).

### Statistical analyses

Statistical analyses of behavioural evaluations, serum parameters, and body composition parameters were performed using PASW Statistics version 21 (SPSS Inc., USA), for p ≤ 0.05. Comparisons among the four groups were performed by two-way ANOVA followed by Tukey HSD test, adopting ovariectomy and GbE treatment as the fixed factors.

Interactions between behavioural parameters and body composition/serum measurements were assessed by the Pearson's method and the correlation coefficients were classified as null (r = 0), weak (0 < r ≤ 0.3), moderate (0.3 < r ≤ 0.7) and strong (0.7 < r ≤ 1). Statistical significances were set at *p* < 0.05.

Linear regression analysis was used to identify body composition and serum parameters significantly influencing behavioural variables. The regression models were constructed based on the statistically significant correlations shown by the univariate analysis. Both Pearson’s correlation coefficient and linear regression analysis were performed using STATISTICA 12.0 (StatSoft, Tulsa, OK, USA).
